# Manta Ray Foraging Optimization Transfer Learning-Based Gastric Cancer Diagnosis and Classification on Endoscopic Images

**DOI:** 10.3390/cancers14225661

**Published:** 2022-11-17

**Authors:** Fadwa Alrowais, Saud S. Alotaibi, Radwa Marzouk, Ahmed S. Salama, Mohammed Rizwanullah, Abu Sarwar Zamani, Amgad Atta Abdelmageed, Mohamed I. Eldesouki

**Affiliations:** 1 Department of Computer Sciences, College of Computer and Information Sciences, Princess Nourah bint Abdulrahman University, P.O. Box 84428, Riyadh 11671, Saudi Arabia; 2 Department of Information Systems, College of Computing and Information System, Umm Al-Qura University, Saudi Arabia; 3 Department of Information Systems, College of Computer and Information Sciences, Princess Nourah bint Abdulrahman University, P.O. Box 84428, Riyadh 11671, Saudi Arabia; 4 Department of Electrical Engineering, Faculty of Engineering & Technology, Future University in Egypt, New Cairo 11845, Egypt; 5 Department of Computer and Self Development, Preparatory Year Deanship, Prince Sattam bin Abdulaziz University, AlKharj, Saudi Arabia; 6 Department of Information System, College of Computer Engineering and Sciences, Prince Sattam bin Abdulaziz University, AlKharj, Saudi Arabia

**Keywords:** gastric cancer, medical diagnosis, deep learning, transfer learning, endoscopic images

## Abstract

**Simple Summary:**

This paper aims to develops a new Manta Ray Foraging Optimization Transfer Learning technique that is based on Gastric Cancer Diagnosis and Classification (MRFOTL-GCDC) using endoscopic images.

**Abstract:**

Gastric cancer (GC) diagnoses using endoscopic images have gained significant attention in the healthcare sector. The recent advancements of computer vision (CV) and deep learning (DL) technologies pave the way for the design of automated GC diagnosis models. Therefore, this study develops a new Manta Ray Foraging Optimization Transfer Learning technique that is based on Gastric Cancer Diagnosis and Classification (MRFOTL-GCDC) using endoscopic images. For enhancing the quality of the endoscopic images, the presented MRFOTL-GCDC technique executes the Wiener filter (WF) to perform a noise removal process. In the presented MRFOTL-GCDC technique, MRFO with SqueezeNet model is used to derive the feature vectors. Since the trial-and-error hyperparameter tuning is a tedious process, the MRFO algorithm-based hyperparameter tuning results in enhanced classification results. Finally, the Elman Neural Network (ENN) model is utilized for the GC classification. To depict the enhanced performance of the presented MRFOTL-GCDC technique, a widespread simulation analysis is executed. The comparison study reported the improvement of the MRFOTL-GCDC technique for endoscopic image classification purposes with an improved accuracy of 99.25%.

## 1. Introduction

Gastric cancer (GC) is the fifth most common cancer across the globe and the third leading factor of tumor death [1]. There is an extensive geographic variance in its prevalence, with the maximum occurrence rate being in East Asian countries. In China, almost 498,000 new cases of GC have been identified in 2015, and here, it is the 2nd leading factor of cancer-related deaths. As surgical intervention, prior detection, and precise analysis are the decisive elements to reduce the GC death rates, robust and reliably actual pathology services are necessary [2]. However, there is a lack of anatomical diagnosticians globally and nationally, which has formed over-loaded workers, therefore affecting their diagnostic precision. A rising number of pathology labs have implemented digital slides in the form of whole slide images (WSI) in regular diagnostics [3]. The alteration of the practices from microscopes to WSIs has laid the foundation for utilizing artificial intelligence (AI)-guided mechanisms in pathology treatments to address the human limits and minimize the diagnostic faults [4]. This has permitted the growth of new techniques, such as AI through deep learning. The research has concentrated on formulating techniques that could flag suspicious zones, urging pathologists to scrutinize the tissue completely at high magnifications or by using immunohistochemical (IHC) test if they are needed to accomplish a precise analysis [5].

Radiotherapists have started to utilize this technology for reading medical images for several ailments with the growth of AI [6]. AI has a set of inter-related practical methods which overlap the fields of statistics and mathematics, and mathematical functions are considered to be appropriate for radiology due to the pixel values of an MRI image which are computable. Artificial neural networks (ANNs), for example, are one of the methods that is utilized in the sub-discipline of classifier mechanisms [7]. The ideology of deep learning (DL) has garnered substantial interest in ANNs. Several sorts of sub-techniques considering the advancements in memory enhancement, fast processing, and novel model features and models have been constantly upgraded and developed [8]. The common ANN that is utilized by DL is the convolutional neural network (CNNs), which is the most suitable neural network (NNs) for radiology when the images are the main units of evaluation [9]. A CNN can be biologically inspired networks which mimic the brain cortex behavior, which has a complicated structure of cells that are sensitive to smaller areas of the visual domain [10]. The CNN does not just contain a sequence of layers which would map image inputs into desirable end points, it also studies high-level imaging features.

This study focuses on the development of the new Manta Ray Foraging Optimization Transfer learning-based Gastric Cancer Diagnosis and Classification (MRFOTL-GCDC) method using endoscopic images. The presented MRFOTL-GCDC technique executes the Wiener filter (WF) to achieve a noise removal process. Moreover, the MRFOTL-GCDC technique makes use of the SqueezeNet model to derive the feature vectors, and the MRFO algorithm is exploited as a hyperparameter optimizer. Furthermore, the Elman Neural Network (ENN) method was utilized for the GC classification. For ensuring the improvised performance of the presented MRFOTL-GCDC method, a widespread simulation analysis has been carried out.

## 2. Related Works

In [11], a noble openly accessible Gastric Histopathology Sub-size Image Database (GasHisSDB) was established for identifying the classifier outcomes. For proving that the techniques of distinct periods during the domain of image classifiers were discrepant when they were using GasHisSDB, the authors chose a variety of classifications for the calculation. Seven typical ML techniques, three CNN techniques, and a new transformer-based classification were selected to test on image classifier task. Sharanyaa et al. [12] concentrated on developing a robust predictive system which utilizes an image processing approach for detecting the initial stage of cancer with lightweight approaches. The testing images in the pathology dataset termed the BioGPS were pre-processed primarily to remove the noisy part of the pixels. This was realized in deep Color-Net (Deep CNET) technique which relates the trained vector with a testing vector to determine a maximal correlation. With a superior match score, the classifier outcomes defines the occurrence of GC and emphasizes the spread region in the provided test pathology data.

Qiu et al. [13] intended to improve the performance of GC analysis, thus, the DL techniques were tentatively utilized for supporting doctors in the analysis of GC. The lesion instances in the images were each noticeable by several endoscopists who had several years of medical experience. Afterward, the gained trained set was used as an input for the CNN to train on, and at last, they obtained the technique DLU-Net. In [14], a fully automated system was executed to distinguish between the differentiated or undistinguished and non-mucinous or mucinous cancer varieties from a GC tissue whole-slide image in the Cancer Genome Atlas (TCGA) stomach adenocarcinoma database (TCGA-STAD). Valieris et al. [15] examined an effectual ML technique that could forecast DRD in a histopathological image (HSI). The efficacy of our technique is demonstrated by assuming the recognition of MMRD and HRD in breast and GC tissues, correspondingly.

Meier et al. [16] examined the novel approaches for predicting the risk for cancer-specified death in the digital image of immunohistochemically (IHC) stained tissue microarrays (TMAs). Especially, the authors estimated a cohort of 248 GC patients utilizing CNNs in an end-to-end weakly supervised system which was self-determined by a particular pathologist. For the account of the time-to-event features of the output data, the authors established novel survival techniques for guiding the trained network. An et al. [17] intended to validate and train real-time FCNs to allocate a resection margin of early GC (EGC) in indigo carmine chromoendoscopy (CE) or white light endoscopy (WLE), and they estimated their efficiency and that of the magnifying endoscopy with narrow-band imaging (ME-NBI). The authors gathered the CE and WLE images of the EGC lesions to train the FCN technique in ENDOANGEL. From the literature, it is apparent that the existing approaches do not concentrate on the hyperparameter selection process which primarily affect the performance of the classification models. Specifically, the hyperparameters such as the epoch count, batch size, and learning rate selection become important when one is trying to accomplish an improved performance. As the manual trial-and-error technique for hyperparameter tuning is a tiresome and erroneous process, metaheuristic algorithms can be applied. Therefore, in this work, we employ an MRFO algorithm for the parameter selection of the SqueezeNet model.

## 3. The Proposed Model

**Figure 1:**
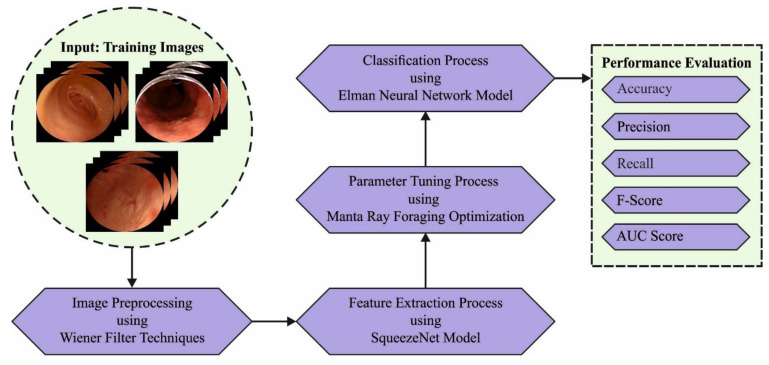
Block diagram of MRFOTL-GCDC system.

In this study, an automated GC classification using an MRFOTL-GCDC technique has been developed for endoscopic images. The presented MRFOTL-GCDC technique exploited the endoscopic images for GC classifications to be made. To accomplish this, the MRFOTL-GCDC technique encompasses the image pre-processing, the SqueezeNet feature extraction, the MRFO hyperparameter tuning, and the ENN classification. Figure 1 defines the block diagram of the MRFOTL-GCDC system.

### 3.1. Stage I: Pre-Processing

In the beginning, the presented MRFOTL-GCDC technique exploited the WF technique to perform a noise eradication process. Noise elimination can be referred as an image pre-processing method which intends to improvise the attributes of the image which has been corrupted through noise [18]. The specific case will be an adaptive filter where the denoising process was reliant on the noise content in the image, locally. Assuming that the images which are corrupted were denoted as $\hat{I}\left(x,y\right)$, the noise variance over whole has been represented as $\sigma_{y}^{2},$ the local mean can be represented as $\hat{\mu_{L}}$ regarding the pixel window, and the local variance from the window was rendered by ${\hat{\sigma }}_{y}^{2}$. Then, the probable method of denoising an image is exhibited below:

(1) $$\hat{\hat{I}}=\hat{I}\left(x,y\right)-\frac{\sigma_{y}^{2}}{{\hat{\sigma }}_{y}^{2}}\left(\hat{I}\left(x,y\right)-\hat{\mu_{L}}\right)\;$$

At this point, if the noise variance across the image was equivalent to 0, $\sigma_{y}^{2}=0=>\hat{\hat{I}}=\hat{I}\left(x,y\right)$. If the global noise variance was less than this, and local variance was more than the global variance, the ratio was nearly equivalent to 1. If ${\hat{\sigma }}_{y}^{2}\gg \sigma_{y}^{2}$, then $\hat{\hat{I}}=\hat{I}\left(x,y\right)$. It was assumed that a higher local variance exemplifies the presence of the edge from the image window. During this case, if the global and local variances were matching, then the formula formulates $\hat{\hat{I}}=\hat{\mu_{L}}\;as\;{\hat{\sigma }}_{y}^{2}\approx \sigma_{y}^{2}$.

### 3.2. Stage II: Feature Extraction

**Figure 2:**
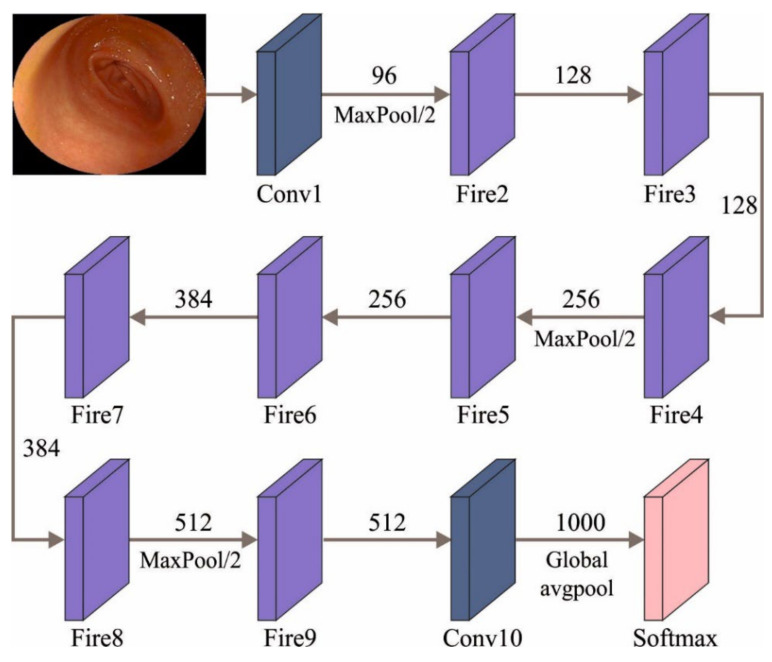
Structure of SqueezeNet model.

At this stage, the MRFOTL-GCDC technique has utilized the SqueezeNet model for the feature extraction. Squeezenet is a type of DNN that has eighteen layers and can be mainly used in computer vision (CV) and image processing programs [19]. The main aims and the purposes of the authors, in the progression of SqueezeNet, were to frame the small NN that has some variables and to perform an easy transmission over the computer network (necessitating minimal bandwidth). Additionally, it must also fit into computer memory, effortlessly (necessitating minimum memory). The primary edition of this structure has been accomplished on top of a DL method that is named Caffe. After a while, the researchers started to use this structure in several publicly available DL structures. Initially, SqueezeNet was labelled, where it is compared with AlexNet. Both SqueezeNet and AlexNet were two distinct DNN structures until now, and they have one common feature, which is termed precision, whenever they are predicting the ImageNet image dataset. The main goal of SqueezeNet was to reach a higher accuracy level while utilizing fewer variables. To achieve this, three processes were employed. Mainly, a filter of size 3 × 3 was replaced by a filter of size 1 × 1 with fewer variables. Then, the number of input channels was minimized to 3 × 3 filters. Lastly, the subsampled function was executed at the final stages to obtain a convolution layer which had a large activation function. SqueezeNet can depend on the idea of an Inception component module for devising a Fire component that has expansion and squeeze layers. Figure 2 establishes the architecture of SqueezeNet method.

In this study, the MRFOTL-GCDC technique designed the MRFO algorithm for the parameter tuning. Zhao et al. [20] proposed an MRFO that was inspired by the foraging approach of a giant marine creature named a Manta ray which are shaped like a bird. This initializes a population of candidate solutions, similar to how Manta rays individually search for better locations. The plankton is focused on; the best solution attained at any point represents the plankton. The search process comprises three stages: somersault foraging, cyclone foraging and chain foraging.

#### 3.2.1. Chain Foraging Phase

In the chain foraging process, each fish in the Manta rays’ school follow the front individual by moving in foraging chain and a better solution has not been found until now. The mathematical formula for chain foraging can be given below:

(2) $$x_{i}^{t+1}=\left\{\begin{array}{l} x_{i}^{t}+r\left(x_{b}-x_{i}^{t}\right)+a\left(x_{b}-x_{i}^{t}\right)\to i=1\\ x_{i}^{t}+r\left(x_{i-1}^{t}-x_{i}^{t}\right)+a\left(x_{b}-x_{i}^{t}\right)\to i=2,\;\ldots ,\;N \end{array}\right.\;$$

(3) $$a=2r\sqrt{\left|\;\log\;\left(r\right)\right|}$$

where $x_{i}^{t}$ indicates the i-th individual location at the iteration $\left(t\right),$ r denotes the random vector belongs to zero and one, and $x_{b}$ signifies the better location that has been attained so far. The upgraded location $\left(x_{i}^{t+1}\right)$ can be implemented using the existing location $\left(x_{i}^{t}\right)$ and the preceding location $\left(x_{i-1}^{t}\right)$ and the better location.

#### 3.2.2. Cyclone Foraging

The Manta ray individual creates a foraging chain and makes a spiral movement when it searches for food sources. In this step, flocked Manta rays pursue the Manta ray that faces the chain and chase the spiral pattern to approach the prey. This spiral motion of the Manta ray in terms of its behavior in the n dimension search space can be mathematically devised below:

(4) $$x_{i}^{t+1}=\left\{\begin{array}{l} x_{b}+r\left(x_{b}-x_{i}^{t}\right)+B\cdot \left(x_{b}-x_{i}^{t}\right)\to i=1\\ x_{b}+r\left(x_{i-1}^{t}-x_{i}^{t}\right)+B\cdot \left(x_{b}-x_{i}^{t}\right)\to i=2,\;\ldots \;,\;N \end{array}\right.$$

(5) $$B=2\;\exp\;\left(r_{1}\cdot \frac{T-t+1}{T}\right)\cdot \;\sin\;\left(2\pi r_{1}\right),\;$$

where B indicates the weight coefficient, T denotes the overall iteration count, and r, $r_{1}\in \left[0,\;1\right]$ characterize a random number. Cyclone foraging allows for the individual Manta rays to use the potential area and obtain a better solution [21]. Furthermore, for better exploration, every individual was forced to discover a novel location that was located farther from its existing location by allocating a reference location that was randomly determined as follows:

(6) $$x_{i}^{t+1}=\left\{\begin{array}{l} x_{rand}+r\left(x_{rand}-x_{i}^{t}\right)+B\cdot \left(x_{rand}-x_{i}^{t}\right)\to i=1\\ x_{rand}+r\left(x_{i-1}^{t}-x_{i}^{t}\right)+B\cdot \left(x_{rand}-x_{i}^{t}\right)\to i=2,\ldots ,N\; \end{array}\right.$$

(7) $$x_{rand}=l_{j}+r\cdot \left(u_{j}-l_{j}\right),\;$$

From the expression, $x_{rand}$ denotes a random location that was indiscriminately located constrained using the lower and upper limits $u_{i}$ and $l_{i}$, correspondingly.

#### 3.2.3. Somersault Foraging

Each Manta ray individually swims backward and forward to pivot to upgrade its position by somersaulting around the better location that was attained in the following:

(8) $$x_{i}^{t+1}=x_{i}^{t}+\psi \left(r_{2}x_{b}-r_{3}x_{i}^{t}\right)\to i=1,\ldots ,N,\;$$

where ψ, which is named as the somersault component, defines the range of the somersault where the Manta ray can swim $\left(\psi =2\right),$ $r_{2}$ and $r_{3}$ represent the random values that lie in between zero and one. Thus, the behaviors of somersault foraging allow for the Manta ray to freely move in a novel domain amongst the position and symmetrical position that is based on the better location. As well, the somersault range was proportionate to the iteration since it decreases as the iteration rises.

### 3.3. Stage III: GC Classification

Finally, the MRFOTL-GCDC technique has utilized the ENN model for classification purposes. The ENN technique includes hidden, input, context, and output layers [22]. The major configuration of the ENN method can be comparable to the FFNN, wherein the connection except context layer is same as the MLP. The context layer obtains inputs from the outputs of the hidden unit to store the earlier value of the hidden unit. The output weight, the external input, and the context weight matrixes were denoted as $W_{h}^{i},$ $W_{h}^{c}$ and $W_{h}^{0}$, correspondingly. The output and input dimension layers are characterized by n, i.e., the dimension of the context layer was m  and $\;x^{1}\left(t\right)={\left[x_{1}^{1}\left(t\right),x_{2}^{1}\left(t\right),\ldots .,x_{n}^{1}\left(t\right)\right]}^{T}$, $y\left(t\right)={\left[y_{1}\left(t\right),\;y_{2}\left(t\right),\;\ldots ,\;y_{n}\left(t\right)\right]}^{T}$.

The input unit of the ENN can be defined using the subsequent formula:

(9) $$u_{i}\left(l\right)=e_{i}\left(l\right),\;i=1,2,\;\ldots ,\;n\;$$

Now, l defines the input and output units at l round. Next, k-th hidden unit in the network is shown below:

(10) $$\begin{array}{c} v_{k}\left(l\right)=\overset{N}{\underset{j=1}{\sum }}\omega_{kj}^{1}\left(l\right)x_{j}^{c}\left(l\right)+\overset{n}{\underset{i=1}{\sum }}\omega_{ki}^{2}\left(l\right)u_{i}\left(l\right)\\ k=1,2,\;\ldots ,\;N\; \end{array}\;$$

Here, $x_{j}^{c}\left(l\right)$ defines the signal viz., which are distributed from the k-th context nodes, $\omega_{kj}^{1}\left(l\right)$ describes i-th and j-th weights of the hidden state directed from o-th node. Lastly, the outcome of hidden unit is fed into the context layer that is given below:

(11) $$W_{k}\left(l\right)=f_{0}\left({\bar{v}}_{k}\left(l\right)\right)\;$$

Now,

(12) $${\bar{v}}_{k}\left(l\right)=\frac{v_{k}\left(l\right)}{\max\left\{v_{k}\left(l\right)\right\}}$$

The abovementioned formula denotes the normalized value of the hidden unit. The succeeding layer represents the context layer as follows:

(13) $$C_{k}\left(l\right)=\beta C,\left(l-1\right)+W_{k}\left(l-1\right),\;k=1,2,\;\ldots ,\;N$$

From the expression, $W_{k}$ denotes the gain of the self-connected feedback [0, 1]. Lastly, the output unit in the network was denoted by:

(14) $$y_{0}\left(l\right)=\overset{N}{\underset{k=1}{\sum }}\omega_{ok}^{3}\left(l\right)W_{k},\left(l\right),\;0=1,2,\;\ldots ,\;n\;$$

From the expression, $\omega_{ok}^{3}$ defines the weight connected from k-th into o-th layers.

## 4. Results and Discussion

**Figure 3:**
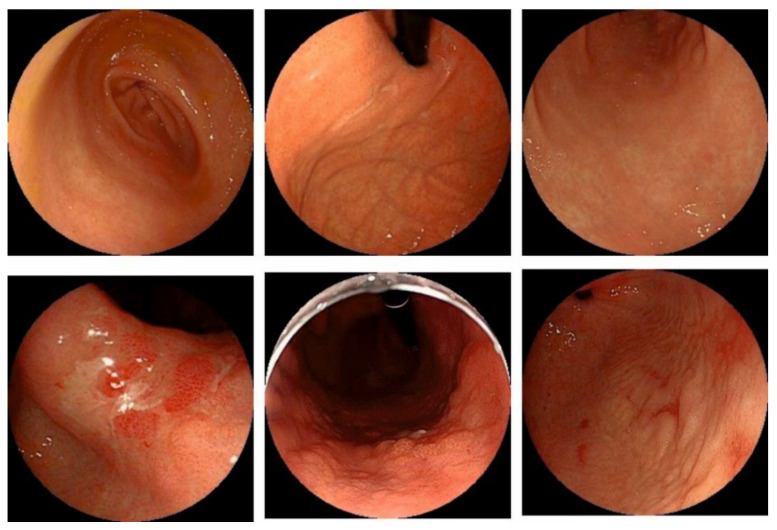
Sample images.

In this section, the GC classification results of the MRFOTL-GCDC method were tested using a dataset that was comprised of a set of endoscopic images. The dataset holds 2377 endoscopy images with three classes, as represented in Table 1. Figure 3 depicts some of the sample images.

**Figure 4:**
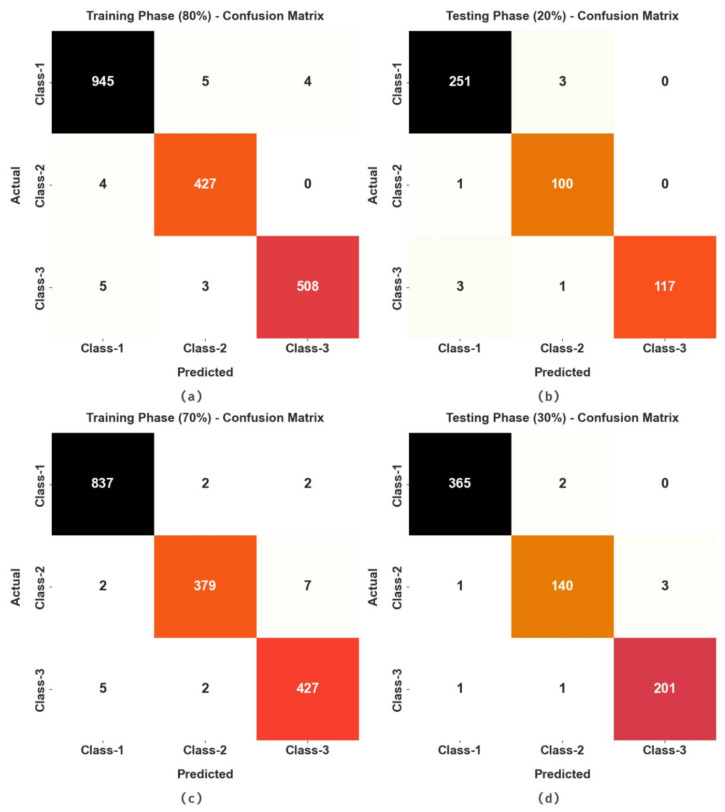
Confusion matrices of MRFOTL-GCDC system (**a**,**b**) TR and TS database of 80:20 and (**c**,**d**) TR and TS database of 70:30.

The confusion matrices which were obtained by the MRFOTL-GCDC method using the GC classification process are shown in Figure 4. The results highlighted that the MRFOTL-GCDC method has properly differentiated the presence of GC.

Table 2 portrays an overall GC classification outcomes of the MRFOTL-GCDC method using 80% of the TR databases and 20% of the TS databases.

**Figure 5:**
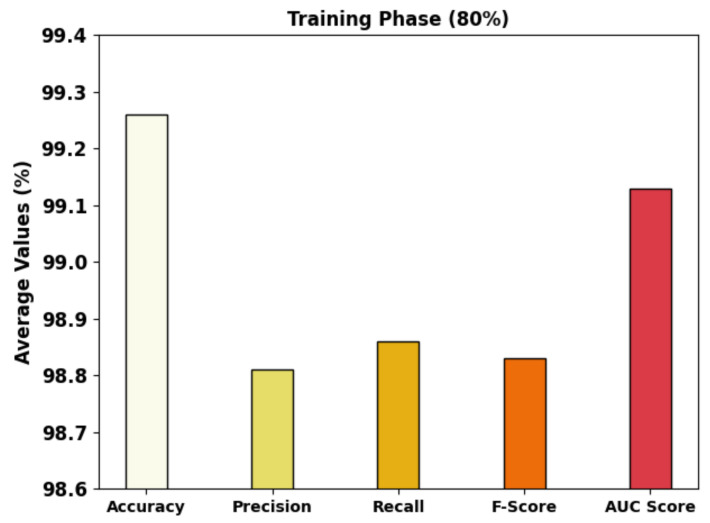
Average analysis of MRFOTL-GCDC system in 80% of the TR database.

Figure 5 exhibits the brief GC classifier outcomes of the MRFOTL-GCDC method using 80% of the TR database. The results exhibit that the MRFOTL-GCDC method has properly differentiated the images into three classes. The MRFOTL-GCDC model has attained an average $accu_{y}$ of 99.26%, a $prec_{n}$ of 98.81%, a $reca_{l}$ of 98.86%, an $F_{score}$ of 98.83%, and an $AUC_{score}$ of 99.13%.

**Figure 6:**
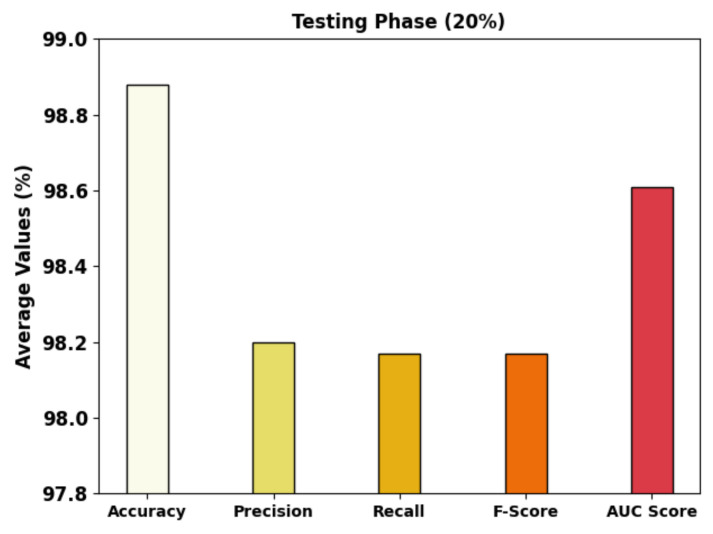
Average analysis of MRFOTL-GCDC system using 20% of the TS database.

Figure 6 portrays the detailed GC classifier outcomes of the MRFOTL-GCDC method using 20% of the TS database. The results that were produced by the MRFOTL-GCDC approach has properly distinguished the images into three classes. The MRFOTL-GCDC method has obtained an average $accu_{y}$ of 98.88%, a $prec_{n}$ of 98.20%, a $reca_{l}$ of 98.17%, an $F_{score}$ of 98.17%, and an $AUC_{score}$ of 98.61%.

**Figure 7:**
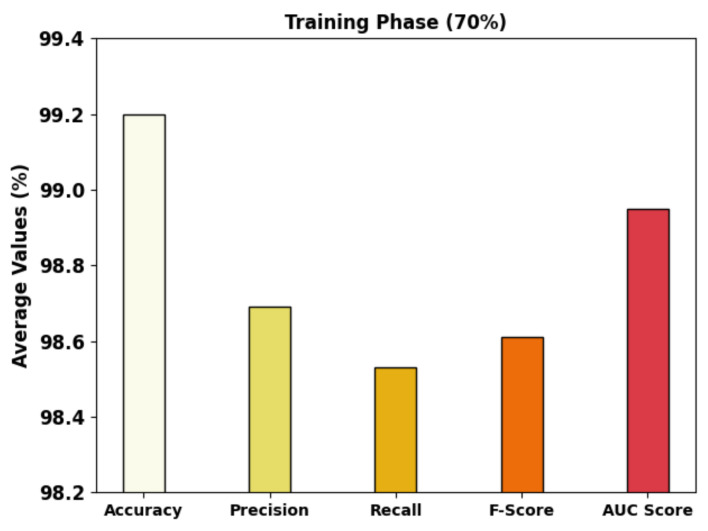
Average analysis of MRFOTL-GCDC system using 70% of the TR database.

Table 3 depicts the overall GC classification outcomes of the MRFOTL-GCDC approach using 70% of the TR databases and 30% of the TS databases. Figure 7 exhibitions the brief GC classifier outcomes of the MRFOTL-GCDC method using 70% of the TR database. The results produced by the MRFOTL-GCDC method have properly distinguished the images into three classes. The MRFOTL-GCDC technique has achieved an average $accu_{y}$ of 99.20%, a $prec_{n}$ of 98.69%, a $reca_{l}$ of 98.53%, an $F_{score}$ of 98.61%, and an $AUC_{score}$ of 98.95%.

**Figure 8:**
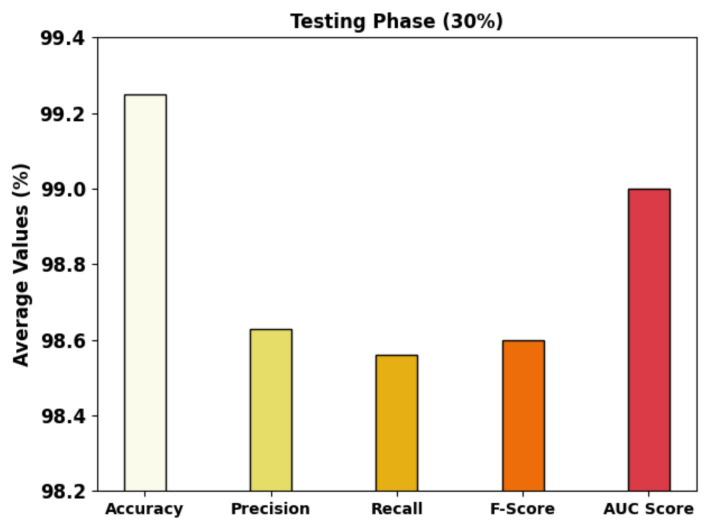
Average analysis of MRFOTL-GCDC system using 30% of the TS database.

Figure 8 displays the complete GC classifier results of the MRFOTL-GCDC approach using 30% of the TS database. The results that were produced by the MRFOTL-GCDC approach have properly distinguished the images into three classes. The MRFOTL-GCDC method has achieved an average $accu_{y}$ of 99.25%, a $prec_{n}$ of 98.63%, a $reca_{l}$ of 98.56%, an $F_{score}$ of 98.60%, and an $AUC_{score}$ of 99%.

**Figure 9:**
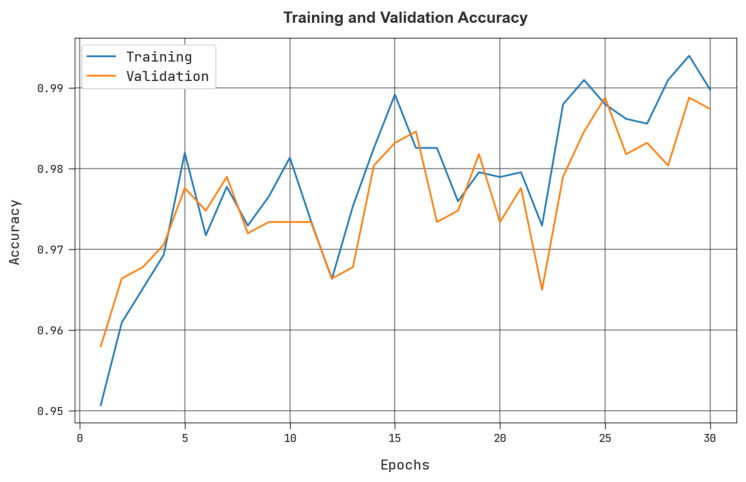
$TR_{acc}$ and $VL_{acc}$ analysis of MRFOTL-GCDC system.

The training accuracy ($TR_{acc}$) and validation accuracy ($VL_{acc}$) that were acquired by the MRFOTL-GCDC approach in the test dataset are shown in Figure 9. The simulation values that were produced by the MRFOTL-GCDC method have reached higher values of $TR_{acc}$ and $VL_{acc}$. Mainly, the $VL_{acc}$ is greater than the $TR_{acc}$ is.

**Figure 10:**
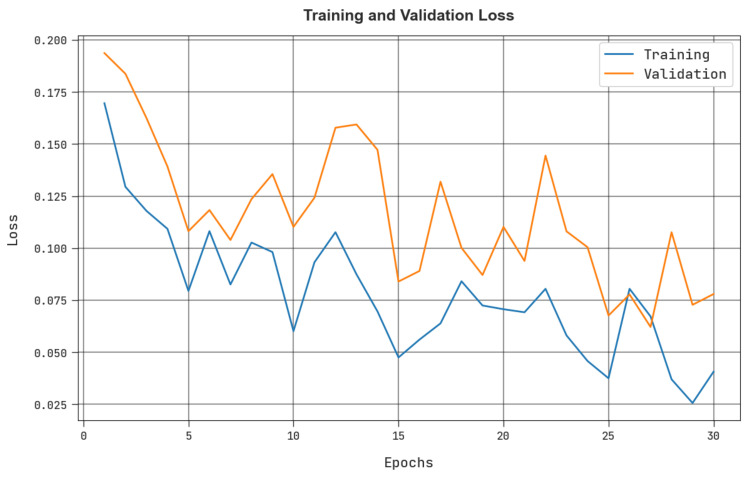
$TR_{loss}$ and $VL_{loss}$ analysis of MRFOTL-GCDC system.

The training loss ($TR_{loss}$) and validation loss ($VL_{loss}$) that were attained by the MRFOTL-GCDC technique in the test dataset are established in Figure 10. The simulation values denoted that the MRFOTL-GCDC approach has exhibited minimal values of $TR_{loss}$ and $VL_{loss}$. Mostly, the $VL_{loss}$ is lower than the $TR_{loss}$ is.

**Figure 11:**
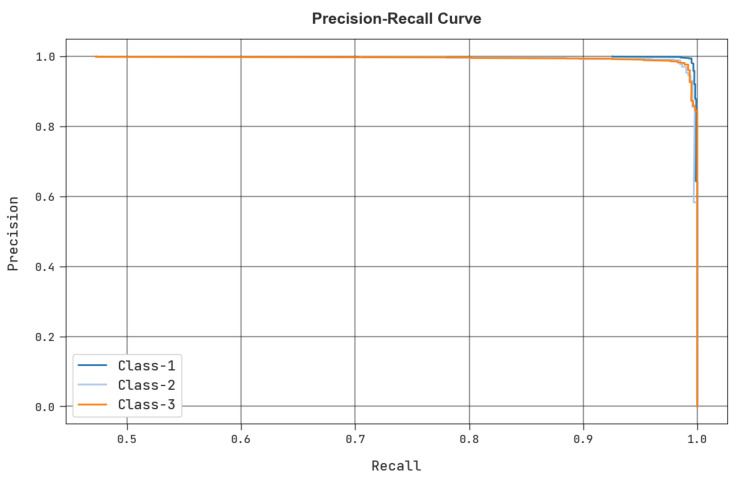
Precision recall analysis of MRFOTL-GCDC system.

A clear precision-recall review of the MRFOTL-GCDC method using the test database is shown in Figure 11. The figure shows that the MRFOTL-GCDC approach has resulted in enhanced values for the precision-recall values in every class.

**Figure 12:**
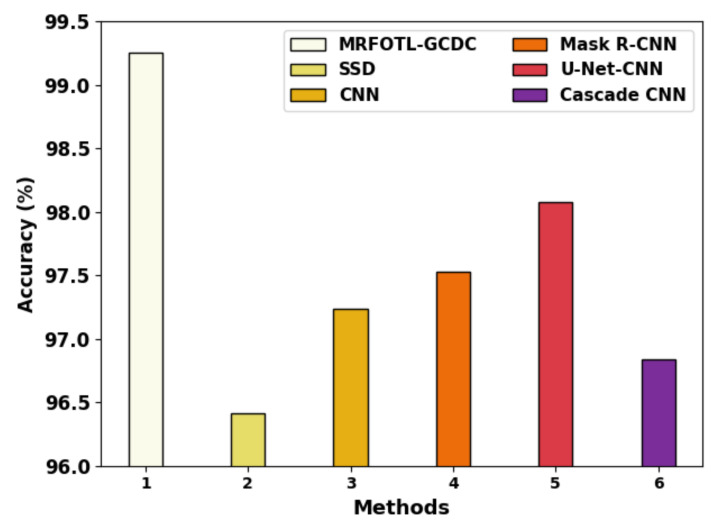
$Accu_{y}$ analysis of MRFOTL-GCDC system with other existing approaches.

Table 4 provides detailed GC classification results of the MRFOTL-GCDC model with recent models. Figure 12 reports comparative results of the MRFOTL-GCDC method in terms of the $accu_{y}$. Based on the $accu_{y}$, the MRFOTL-GCDC model has shown increased the $accu_{y}$ to 99.25%, whereas the SSD, CNN, Mask R-CNN, U-Net-CNN, and cascade CNN models have reported reduced $accu_{y}$ values of 96.41%, 97.24%, 97.53%, 98.08%, and 96.84% correspondingly.

**Figure 13:**
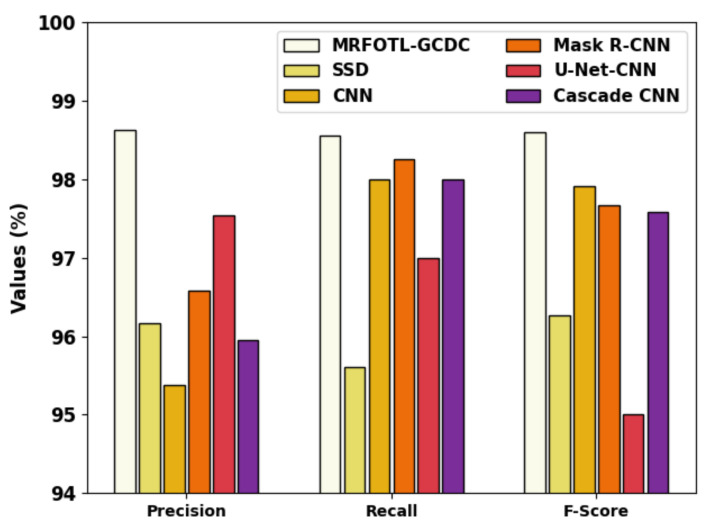
Comparative analysis of MRFOTL-GCDC system with other existing approaches.

Figure 13 exhibits the comparative results of the MRFOTL-GCDC technique in terms of the $prec_{n}$, $reca_{l}$, and $F_{score}$. Based on the $prec_{n}$, the MRFOTL-GCDC approach has displayed an increased $prec_{n}$ at 98.63%, whereas the SSD, CNN, Mask R-CNN, U-Net-CNN, and cascade CNN techniques have reported reduced $prec_{n}$ values of 96.16%, 95.38%, 96.58%, 97.54%, and 95.95% correspondingly. Additionally, based on the $reca_{l}$, the MRFOTL-GCDC model has shown increased $reca_{l}$ at, 98.56% whereas the SSD, CNN, Mask R-CNN, U-Net-CNN, and cascade CNN models have reported reduced $reca_{l}$ values of 95.61%, 98%, 98.25%, 96.99%, and 98% correspondingly.

Finally, based on the $F_{score}$, the MRFOTL-GCDC approach has shown an increased $F_{score}$ of 98.60%, whereas the SSD, CNN, Mask R-CNN, U-Net-CNN, and cascade CNN models have reported reduced $F_{score}$ values of 96.26%, 97.91%, 97.67%, 95%, and 97.58% correspondingly. These results reported the improvement of the MRFOTL-GCDC model.

## 5. Conclusions

In this study, an automated GC classification using the MRFOTL-GCDC technique has been developed for endoscopic images. The presented MRFOTL-GCDC technique examined the endoscopic images for the identification of GC using DL and metaheuristic algorithms. The presented MRFOTL-GCDC technique encompasses WF based preprocessing, SqueezeNet feature extraction, MRFO hyperparameter tuning, and ENN classification techniques. The experimental result analysis of the MRFOTL-GCDC technique demonstrates the promising endoscopic image classification performance with a maximum accuracy of 99.25%. In future, the detection rate of the MRFOTL-GCDC technique can be boosted by deep instance segmentation and deep ensemble fusion models.

## Figures and Tables

**Table 1:** Dataset details.

Label	Class	Count of Images
Class1	Healthy	1208
Class2	Early gastric cancer	532
Class3	Advanced gastric cancer	637
**Total No. of Images**		**2377**

**Table 2:** Result analysis of MRFOTL-GCDC system at an 80:20 ratio of the TR/TS database.

Labels	$Accu_{y}$	$Prec_{n}$	$reca_{l}$	$F_{score}$	AUC Score
Training Phase (80%)					
Class-1	99.05	99.06	99.06	99.06	99.05
Class-2	99.37	98.16	99.07	98.61	99.26
Class-3	99.37	99.22	98.45	98.83	99.08
**Average**	**99.26**	**98.81**	**98.86**	**98.83**	**99.13**
**Testing Phase (20%)**					
Class-1	98.53	98.43	98.82	98.62	98.51
Class-2	98.95	96.15	99.01	97.56	98.97
Class-3	99.16	100.00	96.69	98.32	98.35
**Average**	**98.88**	**98.20**	**98.17**	**98.17**	**98.61**

**Table 3:** Result analysis of MRFOTL-GCDC system at a 70:30 ratio of TR/TS database.

Labels	$Accu_{y}$	$Prec_{n}$	$reca_{l}$	$F_{score}$	AUC Score
Training Phase (70%)					
Class-1	99.34	99.17	99.52	99.35	99.34
Class-2	99.22	98.96	97.68	98.31	98.68
Class-3	99.04	97.94	98.39	98.16	98.83
**Average**	**99.20**	**98.69**	**98.53**	**98.61**	**98.95**
**Testing Phase (30%)**					
Class-1	99.44	99.46	99.46	99.46	99.44
Class-2	99.02	97.90	97.22	97.56	98.35
Class-3	99.30	98.53	99.01	98.77	99.21
**Average**	**99.25**	**98.63**	**98.56**	**98.60**	**99.00**

**Table 4:** Comparative analysis of MRFOTL-GCDC system with other existing techniques.

Methods	$Accu_{y}$	$Prec_{n}$	$reca_{l}$	$F_{score}$
MRFOTL-GCDC	99.25	98.63	98.56	98.60
SSD	96.41	96.16	95.61	96.26
CNN	97.24	95.38	98.00	97.91
Mask R-CNN	97.53	96.58	98.25	97.67
U-Net-CNN	98.08	97.54	96.99	95.00
Cascade CNN	96.84	95.95	98.00	97.58

## Data Availability

Not applicable.
